# Stability and manoeuvrability in animal movement: lessons from biology, modelling and robotics

**DOI:** 10.1098/rspb.2021.2492

**Published:** 2022-01-26

**Authors:** Andrew A. Biewener, Richard J. Bomphrey, Monica A. Daley, Auke J. Ijspeert

**Affiliations:** ^1^ Concord Field Station, Department of Organismic and Evolutionary Biology, Harvard University; ^2^ Structure and Motion Laboratory, Department of Comparative Biomedical Sciences, The Royal Veterinary College, London, UK; ^3^ Department of Ecology and Evolutionary Biology, University of California, Irvine, CA, USA; ^4^ Institute of Bioengineering, Ecole Polytechnique Fédérale de Lausanne (EPFL), Lausanne, Switzerland

**Keywords:** stability, manoeuvrability, locomotion, robotics, bioengineering, neuromechanics

## 1. Introduction

New technologies and the increasing integration of biological and engineering approaches are revealing fundamental new insights into how animals actuate and control movement to achieve agility, stability and economy while manoeuvring and navigating through complex environments. As a result, new information is emerging about how flying, swimming and running animals rapidly and efficiently generate media-based forces, process multi-modal sensory information and make use of intrinsic neuromechanical mechanisms to achieve robust control of motor behaviours over short time scales. In parallel, new computational approaches provide powerful tools for modelling the neuro-musculoskeletal control of complex body systems with many degrees of freedom. These experimental and modelling approaches, in turn, are driving the development and implementation of diverse bioinspired robots based on abstracted biological concepts, leading to new insights for the rehabilitation of human neuromotor deficits.

Agile locomotion is fundamental to successful biological performance, such as predator–prey interactions, mating, obstacle traversal and avoidance, and navigation, as well as engineering designs that emulate such performance. By including perspectives of locomotion over and through different media, commonalities of approaches across animals emerge that may reflect convergent evolutionary pathways, as well as commonalities of approaches taken by researchers working in these related fields. We summarize below the main results and approaches taken by the authors of the papers published in this theme issue on *Stability and manoeuvrability in animal movement: lessons from biology, modelling and robotics.* The assembled topics cover modes of animal locomotion in water, in air and over ground, highlighting novel interdisciplinary approaches that integrate neuroscience, biomechanics and physics with computational methods and engineering design.

## 2. Aquatic locomotion

In their paper ‘The most efficient metazoan swimmer creates a “virtual wall” to enhance performance’, Gemmell *et al*. [1] show that when swimming steadily the moon jellyfish *Aurelia aurita* produces a stopping vortex (that is absent when swimming from rest), which interacts with the opposite sign starting vortex generated by contraction of its bell. The resulting vortex–vortex interaction beneath the bell's subumbrellar surface results in an effective ‘virtual wall’ created by the convergence of fluid at the vortex interface, which produces significantly greater pressure and thrust leading to a greater swimming speed, without the need to alter swimming kinematics. The presence of a ‘virtual wall’, combined with earlier evidence of ‘passive energy recapture’ [2], enables open water swimming jellyfish to achieve the lowest energy cost of transport for any swimming metazoan. The ‘virtual wall’ effect parallels the well-known ‘ground effect’ previously observed for animals (and machines) swimming and flying above ground, which enhances fluid forces and reduces the energy cost of locomotion. The exceptional swimming efficiency of jellyfish probably underlies their 500 Myr evolutionary success and relates to the fact that they have a single muscle cell layer in the bell, which contracts uniformly due to all-or-none motor activation. As a result, the bell's contraction kinematics are uniform whether the animal starts from rest or when swimming steadily. This unique feature of jellyfish is exploited by Gemmell and colleagues using digital particle image velocimetry to separate and identify the fluid vortex–vortex interaction effects that generate the ‘virtual wall’ and enhanced swimming performance. Similar vortex–vortex interactions that enhance fluid forces have been observed in the wake recapture of flying insects [3]. It seems likely that similar interactions may also occur in the median fins and tail of swimming fishes. Such observations of the evolutionary novelty and success of biological fluid propulsion mechanisms are likely to stimulate the design of more efficient bio-engineered propulsion devices.

In contrast with the moon jellyfish, fast, pelagic, predatory fish such as tuna have a broad ‘kinematospace’ that facilitates impressive agility. While the hydrodynamics of steady-state, cruising swimming in fish can now be measured routinely, it is a greater challenge to determine the fluid forces underpinning unsteady manoeuvring because animal behaviour ensures variation of kinematics and a lack of repeatability. In ‘Tuna robotics: hydrodynamics of rapid linear accelerations', Thandiackal *et al*. [4] sidestepped behavioural variation by using an innovative robotic platform: Tunabot Flex. They commanded the system to accelerate from rest by oscillating with different frequencies. As it did so, they recorded the electrical power consumption of the robot, measured the surrounding flow fields using particle image velocimetry, and then calculated pressure forces around the head, body and tail. They found that the lateral forces and drag on the head are particularly high relative to steady-state swimming, and open new avenues for the study of kinematics and morphology in manoeuvring fish.

## 3. Insect flight control

In Diptera (the true flies), the erstwhile hindwings of ancestral forms have been reduced to specialized mechanosensory organs called halteres. Halteres have slender shafts and bulbous tips, and when they oscillate, they bend out of plane under Coriolis forces if the body rotates. Monitoring the strains provides information about movement that is important for flight control. Halteres also play a role in maintaining the timing of the wingbeat. In their paper, ‘Takeoff diversity in Diptera’, Yarger *et al*. [5] show that, in a relatively recent calyptrate branch of Diptera, the flies use their halteres in another way too: when rapidly extending their legs to take off. Calyptrate flies, including house flies and blowflies but not the genetic model species of *Drosophila*, oscillate their halteres while walking and also use them to control fast take-off behaviour. Using ablation experiments to deprive flies of their halteres, the authors showed diminished take-off speed and body stability in calyptrate flies during and just after leg extension, yet this observation does not apply to non-calyptrate flies. More generally, the paper presents an example of how mechanosensation can be used for stabilization and locomotor control even when key sensors (which in this case are integrated within the halteres) are placed some distance from the principal actuators (the rapidly extending legs during jumping).

As insects fly, sensory information is gathered from a suite of sensor types known to be useful for guidance, navigation and control tasks. Each receptor and neuron has its own properties (sensitivity threshold, frequency response, cost of activation, conduction velocity, etc.), and these may be dependent on anatomical location. At some point in the sensorimotor control system, incoming information from different modes is fused before motor commands are sent to the muscles responsible for power, stabilization or steering. Therefore, single-input, single-output experimental methods are gradually being replaced by experimental frameworks that include multiple inputs. In their paper, ‘Haltere and visual inputs sum linearly to predict wing (but not gaze) motor output in tethered flying *Drosophila*’, Rauscher & Fox [6] devise such an experiment to investigate visual and mechanosensory systems. They stimulated motion vision neurons sensitive to yaw rotations by means of a moving, high-contrast grating and the halteres by the attachment of iron filings in the presence of a changing magnetic field. Their results show that—despite being founded on highly nonlinear physiological characteristics—visual and haltere stimuli sum linearly to predict wing steering responses. However, while the neck motor system that controls gaze direction was also affected by magnetic manipulations of the haltere kinematics, head steering was not well predicted using the same superposition of each sensory mode. This suggests that different control architectures might be at play, despite their integrated roles in flight control.

Insect flight involves extremely fast reactions to perturbations during wing beats. In ‘Timing precision in fly flight control: integrating mechanosensory input with muscle physiology’, Dickerson [7] reviews the role of the underlying mechanosensory feedback from wings and halteres during flight in flies. The feedback loops to steering muscles are so fast (at sub-millisecond time scales) that they depend on the exact timings of single spikes. The feedback signals are synchronous with the wingbeats and are active at frequencies of the order of hundreds of hertz depending on the fly species. Dickerson reviews evidence that halteres serve several roles: as metronomes and as gyroscopic sensors (i.e. in responding to body rotations that induce Coriolis forces). He also proposes that flies do not use halteres as passive sensors in fixed-gain feedback loops, but that they actively tune the strength of haltere feedback to achieve voluntary flight turns. In this *control loop hypothesis,* visual commands modulate the feedback strength by activating haltere muscles, which results in changes of flight direction. Evidence from the blowfly *Calliphora vicina* and the fruit fly *Drosophila melanogaster* support that hypothesis. Hence the haltere should be viewed as a multifunctional sensory organ that is involved both in maintaining stability in the face of external perturbations but also in visually guided voluntary manoeuvres. The nested control loop design Dickerson [7] proposes (a vision loop nested within a haltere loop) shares similar features as that proposed by Rauscher & Fox [6], which involve interactions of visual and haltere-based mechanosensory inputs to control wing steering movements, but also may differ depending on the species studied.

The compound eyes of insects are, of course, multifunctional themselves. Alongside image formation, target tracking, obstacle avoidance and a number of other tasks, the lobula complex of the insect brain functions to process delays in spatial patterns of contrasts moving across the ommatidia in order to estimate self-motion using optic flow. The ventral visual field can be used to determine ground speed or relative changes in altitude for a given spatial pattern, although these two variables are entwined; the angular rate of the visual texture passing beneath a flying insect is sensitive to both, because either reducing airspeed or increasing altitude both slow the rate of optic flow. While bees appear to show an innate preference for a particular angular rate, this can be limited by maximum airspeed (in the case of headwinds) or make flight control problematic (in the case of tailwinds where the airspeed may become negative). This paradigm was elucidated by Baird *et al*. [8] in their paper, ‘The effect of optic flow cues on honeybee flight control in wind’. They conducted a set of experiments to test hypotheses that heights selected by bees should be affected by head- and tailwinds. They found that the honeybees could indeed control flight altitude and speed in predictable ways but also, intriguingly, that lateral motion of the flight trajectory can provide independent assessment of altitude that is not confounded by forward flight speed. This orthogonality of information—despite the non-orthogonal, hexagonal matrix, sensor arrays of ommatidia—can be used by the bees in flight control when inputs are otherwise ambiguous.

Insect flight is energetically demanding and requires precision control to maintain stability. In hawkmoths (*Manduca sexta*), flight downstroke is powered by the synchronous dorso-longitudinal muscles, which typically operate at a consistent frequency to maintain a constant wingbeat frequency. It had previously been hypothesized [9,10] that insects that operate their wings at the resonance of the entire thorax are unlikely to modulate wingbeat frequency on short time scales due to the inherent control constraints and power demands imposed by deviations from the energetically favourable mechanical resonance. In their study, ‘Rapid frequency modulation in a resonant system: aerial perturbation recovery in hawkmoths', Gau *et al*. [11] tested whether hawkmoths modulate wingbeat frequency and amplitude in response to sudden vortex ring perturbations. The authors discovered that hawkmoths were able to modulate both frequency and amplitude on short time scales, with a 32% change in frequency on a single-wingstroke time scale. This demonstrates a higher potential for active wingbeat control than has previously been appreciated for insects that power and control flight through elastic resonance.

Aerial manoeuvrability and righting recovery are essential for prey pursuit and capture in dragonflies. During prey capture, a dragonfly often approaches the prey from below and captures the prey when in an inverted state. In their study, ‘Dragondrop: a novel passive mechanism for aerial righting in the dragonfly’, Fabian *et al*. [12] investigate dragonfly aerial righting manoeuvres by dropping dragonflies from inverted positions and measuring three-dimensional kinematics, recovery times and recovery mechanisms. They discovered that all dragonflies were able to right themselves, showing an inherent preference to perform a pitch-up recovery, using a dihedral wing posture and the long abdomen to provide a passive airframe mechanism for aerial righting. Dragonflies falling up-side down took twice as long to arrest their fall velocity compared to animals falling right-side up and took twice as long to initiate wing movements. This shows that the dragonfly sometimes allows for passive recovery at the cost of a longer recovery time. However, the decision processes involved remain unknown.

## 4. Terrestrial locomotion, robotics and modelling

The problem of how multi-legged animals adapt their gaits depending on terrain properties is addressed by Othayoth *et al*. [13] in their study, ‘Locomotor transitions in the potential energy landscape-dominated regime’. They investigate ‘terradynamic’ principles of locomotor transitions, using simplified model systems representing distinct challenges in complex three-dimensional terrains, for instance squeezing through pillars and flexible beams, crossing gaps, going-over steps or self-righting after flipping over. Interestingly, they introduce the concept of a general potential energy landscape to represent these different locomotor–terrain interactions. The locomotion of animals (e.g. cockroaches) and robots (e.g. hexapedal robots) can be analysed with their approach. Othayoth *et al*. discover that both animals and robots display stereotypical locomotor modes, even though they make stochastic transitions between modes. Specific modes correspond to minima basins of attractions of the potential energy landscape, and transitions can be viewed as destabilizing barrier-crossing transitions between basins. These transitions can be intelligently induced by adjusting self-propulsion against the environment or even merely through the stochastic nature of locomotion. Using active feedback mechanisms, animals can voluntarily switch to more favourable modes that overcome lower potential energy barriers during the entire course of traversal, for instance to traverse beam obstacles. Their approach represents an innovative and interesting way to analyse the neuromechanics of locomotion in animals that takes into account physical properties of the environment. It can also be used to characterize and optimize the locomotion performance of robots [14,15], with the ability to properly adjust locomotion modes to particular terrains.

In their paper, ‘Simple decentralized control mechanism that enables limb adjustment for adaptive quadruped running’, Fukuhara *et al*. [16] develop and demonstrate a conceptual model for interlimb coordination for quadrupedal locomotion based on the principle of decentralized control. Their model is made of oscillators representing rhythmic central pattern generator (CPG) circuits with force and velocity feedback loops, but with the particularity that they do not include direct couplings between oscillators (e.g. between forelimbs and hindlimbs). In this model, the limb trajectory is controlled through a phase oscillator driving the shoulder and hip joints in a planarized quadrupedal model. A model-based analysis of sensory feedback coupling is used as a framework to explain how different gaits emerge from the coupled vertical oscillations of the hindlimbs, forelimbs and trunk. The model analysis suggests that sensing of vertical velocities of each body part is essential for interlimb coordination for periodic behaviour of running and landing (but the neural mechanisms underlying this velocity estimation remain to be elucidated). The fact that stable gaits emerged without direct couplings between oscillators highlights that indirect interactions through sensory feedback can represent an important coordination mechanism between limbs (in addition to, or even instead of, direct couplings within CPG circuits).

In their paper ‘Upper body and ankle strategies compensate for reduced lateral stability at very slow walking speeds', Best & Wu [17] analyse the biomechanics and kinematics of humans walking at very slow speeds (0.1 to 0.6 m s^−1^; normal human walking speeds are approximately 1.2 to 1.4 m s^−1^) to investigate how lateral stability may be compromised due to dynamic effects resulting from changes in centre of mass (CoM) position of the trunk and body relative to the location of the centre of pressure (CoP), exerted by the ground reaction force on the base of the foot. This affects the margin of stability (MoS), defined by the authors as the lateral horizontal distance of the CoM relative to the CoP during each step. Past work had examined human walking at faster and self-selected speeds, but had not focused on very slow speeds, which are more typical of the speeds that elderly subjects use as they age and are associated with increased fall risk owing to improper shifting of body weight support during step transitions. Best & Wu show that minimum MoS decreased with gait speed due to increased lateral excursions of the CoM that were not compensated by lateral shifts in CoP. As a result, ankle eversion and hip abduction torques also increased with slower walking. Changing step width, which is a main strategy of healthy subjects walking at faster speeds, was not observed during slow speed walking. Consequently, increased ankle eversion and hip abduction torque appear to be the key strategies used during very slow walking. Future studies need to examine active versus passive mechanisms based on muscle activity recordings (EMG) as well as control of fore-aft pitch torque, which can also contribute to fall risk.

In her field perspective paper, ‘Human biomechanics perspective on robotics for gait assistance: challenges and potential solutions’, Wu [18] reviews past research related to the key challenges that exist for how to apply current understanding of the fundamental principles of terrestrial locomotion to the development of more effective robotic and exoskeletal assistance of human gait. These challenges include the integration of physical hardware and software control algorithms that can assist impaired gait users to adopt a variety of gaits, as well as a reliable mechanism for evaluating the efficacy and performance of their use of an assistive device. Intuitive shared control between the user and the assistance device is also required, which has had limited success to date. Often high interaction loads exist between the user and the assistance device, particularly if the trajectories of a gait-training device are too restrictive. Wu further notes that it is unclear if gait-training therapies aid human motor recovery. Consequently, ‘assist-as-needed’ and myoelectric (EMG) control may provide improvements for more shared control than simple playback of gait kinematics to control a gait assistance device. One promising approach for spinal cord injury subjects is to use a neuromuscular controller based on reflex-based gait simulation [19] to generate and assist gait. The reflex approach yields a measure of shared control and produces joint torques that are not predefined and emerge from the user's joint kinematics and footfall patterns rather than being tightly prescribed, as is the more traditional approach of robotic gait-trainers. Wu concludes that assistive robots need to provide appropriate gait patterns while remaining adaptable to user movements and intentions. Improved understanding and application of fundamental gait principles based on studies of locomotion across a range of behaviours are likely to prove key to enabling more natural locomotor behaviours achieved through human–robot interactions.

In their paper ‘Perspective on musculoskeletal modelling and predictive simulations of human movement’, De Groote & Falisse [20] provide a comprehensive review of how predictive neuro-musculoskeletal simulations can aid improvements to the assessment and treatment of human gait deficits. ‘Predictive simulations' are a recent advancement over ‘tracking simulations’, which depend on matching the simulation of movement to measured body kinematics. By contrast, the authors argue that predictive simulations can reveal principles of locomotion by elucidating cause–effect relationships of the neuro-musculoskeletal system without always having to rely on experimental data for rigorous experimental validation. At the same time, De Groote & Falisse note, as others have demonstrated [21–23], that over-simplifications of Hill-type muscle models and their contractile properties commonly used in musculoskeletal simulations, as well as the lack of validation of the models [24] and abstraction of neuromotor control beyond spinal reflex control of muscle activation, force and joint torque patterns [19], limit the quality of both predictive and tracking simulations of movement. The authors note that future applications will require simulation approaches that take uncertainty (e.g. sensory noise) into account, in addition to validation studies that demonstrate the ability of simulations to accurately predict gait in novel circumstances. Key insights derived from predictive simulations of movement are (i) the need for further evaluation of the optimality criteria (e.g. metabolic cost, muscle activation intensity, muscle stress and joint acceleration) that are used to model motor coordination and gait, (ii) evaluation of the efficacy of gait control architectures, (iii) how altered neuromuscular properties affect gait due to ageing and impairment, and (iv) that incorporating personalized musculoskeletal models is needed to more effectively translate predictive simulations to the treatment of individual subjects who vary in terms of neuro-musculoskeletal properties and gait performance.

## 5. Conclusion

We are living in exciting times with fast progress on both scientific and engineering fronts. This special issue highlights the beautiful diversity and agility of animal locomotion in different movement modes and through different environmental media. The papers within this issue reveal common principles underlying movement among different animals, including control loops running in parallel, the interplay of feedforward and feedback mechanisms, and the fundamental importance of biomechanics underlying agile and robust behaviour of moving animals. Engineering tools have enabled exciting progress in innovative experimental set-ups, more realistic modelling and improved analysis to help decipher principles of animal locomotion. This theme issue provides a glimpse of how these tools and improved understanding might lead to better robots and more effective assistive devices, such as actuated exoskeletons and prostheses. Despite these advances, many challenges remain. Specifically, future studies will need to address non-steady-state locomotory behaviour and locomotion through a wider range of environmental contexts. Additionally, it is essential to consider movement throughout ontogeny because animals must move throughout growth. Finally, there is a need for greater understanding of motor learning and planning, particularly in the context of unsteady manoeuvring and agility behaviours—animals appear to have the ability to learn quickly from one or a few attempts at a novel behaviour [25], yet this fascinating ability is still not properly deciphered. Such advances in knowledge will help enable agile and robust autonomous robots and assistive devices that can adapt to the specific needs and abilities of the user.

## Data accessibility

This article has no additional data.
